# The RNA Directed DNA Methylation (RdDM) Pathway Regulates Anthocyanin Biosynthesis in Crabapple (*Malus* cv. spp.) Leaves by Methylating the *McCOP1* Promoter

**DOI:** 10.3390/plants10112466

**Published:** 2021-11-15

**Authors:** Yifan Xing, Ziyi Xie, Weilei Sun, Yuying Sun, Zhenyun Han, Shiya Zhang, Ji Tian, Jie Zhang, Yuncong Yao

**Affiliations:** 1 Beijing Advanced Innovation Center for Tree Breeding by Molecular Design, Beijing University of Agriculture, Beijing 102206, China; 201930212015@bua.edu.cn (Y.X.); 202030221142@bua.edu.cn (Z.X.); 201930212009@bua.edu.cn (W.S.); 201930212010@bua.edu.cn (Y.S.); 202030212035@bua.edu.cn (Z.H.); 202030212016@bua.edu.cn (S.Z.); tianji19850331@126.com (J.T.); 2 Department of Plant Science and Technology, Beijing University of Agriculture, Beijing 102206, China

**Keywords:** RNA directed DNA methylation, anthocyanin, *COP1*, crabapple

## Abstract

The synthesis of anthocyanin pigments in plants is known to be regulated by multiple mechanisms, including epigenetic regulation; however, the contribution of the RNA-directed DNA methylation (RdDM) pathway is not well understood. Here, we used bisulfite sequencing and Real Time (RT)-quantitative (q) PCR to analyze the methylation level of the promoter of constitutively photomorphogenic 1 (*McCOP1*) from *Malus* cv. spp, a gene involved in regulating anthocyanin biosynthesis. The CHH methylation level of the *McCOP1* promoter was negatively correlated with *McCOP1* RNA expression, and inhibiting DNA methylation caused decreased methylation of the *McCOP1* promoter and asymmetric cytosine CHH methylation. We observed that the *McCOP1* promoter was a direct target of the RdDM pathway argonaute RISC component 4 (McAGO4) protein, which bound to a *McCOP1* promoter GGTTCGG site. Bimolecular fluorescence complementation (BIFC) analysis showed that RNA-directed DNA methylation (McRDM1) interacted with McAGO4 and another RdDM protein, domains rearranged methyltransferase 2 (McDRM2), to regulate the CHH methylation of the *McCOP1* promoter. Detection of CHH methylation and COP1 gene expression in the *Arabidopsis thaliana* *atago4*, *atdrm2* and *atrdm1* mutants showed that *RDM1* is the effector of the RdDM pathway. This was confirmed by silencing *McRDM1* in crabapple leaves or apple fruit, which resulted in a decrease in *McCOP1* CHH methylation and an increase in *McCOP1* transcript levels, as well as in anthocyanin accumulation. In conclusion, these results show that the RdDM pathway is involved in regulating anthocyanin accumulation through CHH methylation of the *McCOP1* promoter.

## 1. Introduction

Anthocyanins are flavonoid pigments that contribute much of the rich coloration in the plant kingdom, and they are also involved in defense against biotic [1,2,3] and abiotic stresses [4]. Higher anthocyanin levels are also often associated with improved agronomic traits and greater consumer preference and they are recognized human phytonutrients, with benefits as dietary components that include anti-colorectal cancer [5] and gastrointestinal tract cancer [6] properties. Accordingly, there is considerable interest in understanding the molecular processes that affect anthocyanin accumulation.

Anthocyanin biosynthesis is promoted by members of the large MYB transcription factor family, which recognize and bind to response *cis*-elements in the promoter regions of flavonoid biosynthesis genes [7,8,9,10,11,12]. In studies of apple (*Malus domestica*), a correlation was observed between the expression pattern of four MYB genes (*MdMYB1*, *MdMYB10*, *MdMYBA* and *MdMYB110a*) and anthocyanin biosynthesis in fruit peels following exposure to sunlight and low temperatures, both of which elicit anthocyanin accumulation, consistent with a regulatory role in anthocyanin biosynthesis [13,14,15,16]. The *MdMYB10* promoter in red-fleshed apples has six tandem repeats of a *MdMYB10* binding *cis*-element, which allow autoregulation of *MdMYB10* expression and anthocyanin accumulation [14]. Studies have also shown that MdMYB1 interacts with the ubiquitin E3 ligase, MdCOP1, causing it to be ubiquitinated and degraded via the proteasome pathway, providing another mechanism to regulate apple fruit coloration [17]. Additionally, overexpression of pear (*Pyrus communis*) *PbCOP1.1* was found to reduce red coloration in the fruit peel of the ‘Red Bartlett’ pear cultivar [18]. These findings further indicate that *MdCOP1* is involved in ubiquitination and degradation of MdMYB1, thereby affecting anthocyanin biosynthesis. However, the transcriptional regulation of *COP1* in *Malus* crabapple, which is a widely used model for the study of flavonoid biosynthesis, due to its diversely colored germplasm collections and horticultural importance, is still unclear [17,19]. Indeed, crabapple represents an interesting experimental target to test for other potential regulatory systems that might influence anthocyanin accumulation, including potential epigenetic regulation.

DNA methylation represents an epigenetic mechanism for regulating gene expression at the transcription and post-translation levels in eukaryotes [20]. The principle RNA-mediated epigenetic pathway in plants is the RNA Directed DNA Methylation (RdDM) pathway, which mediates de novo DNA methylation of cytosine bases, utilizing small interfering RNAs (siRNAs) [21]. An important element of the RdDM pathway is the DRM2 (DOMAINS REARRANGED METHYLASE 2)-RDM1 (RNA-directed DNA methylation)-AGO4 (argonaute 4) protein complex [22], in which AGO4 binds to small RNAs, including siRNAs or long non-coding RNAs (lncRNAs) [23,24], and cleaves the target RNA transcripts. Moreover, AGO4 catalytic activity is important for the creation of secondary siRNAs that strengthen its repressive effects [25]. DRM2 guides DNA methylation at homologous loci through binding of small RNAs to AGO4 [26,27]. RDM1 binds to single-stranded methylated DNA and controls the production of a 24 nucleotide siRNA, which acts in the RdDM pathway [22]. The RDM1 and AGO4 protein complex regulates the methylation of downstream genes, including ubiquitination genes, MYB-type transcription factors and siRNA regulatory genes, by binding to their promoters [28]. Studies suggest that the RdDM pathway participate widely in various physiological activities in plants, such as seed development, dormancy, fruit ripening, sexual reproduction and, notably for this study, coloration [29,30,31,32,33,34].

A recent study showed that RNA-mediated AGO4 binding to the promoters of certain ubiquitination genes (AT5G26960, AT2G05600, AT3G52030, AT5G41630) directed asymmetric DNA methylation of their genomic regions [28]. Furthermore, it is known that MdAGO4 binds to, and methylates, the *MdMYB1* promoter and thus regulates anthocyanin biosynthesis via the RdDM pathway [35]. We therefore hypothesized that the RdDM pathway coordinates the expression of some ubiqitination genes, thereby indirectly regulating anthocyanin biosynthesis in *Malus* plants.

In this study, the crabapple cultivar ‘Radiant’, which has young leaves and buds that are red to purple and mature leaves that are green, was used to investigate the mechanism by which the RdDM pathway methylates the promoter of *McCOP1* and regulates anthocyanin biosynthesis.

## 2. Results

### 2.1. There Is a Negative Correlation between McCOP1 Promoter Methylation Levels and Transcription in Crabapple Leaves

**Figure 1:**
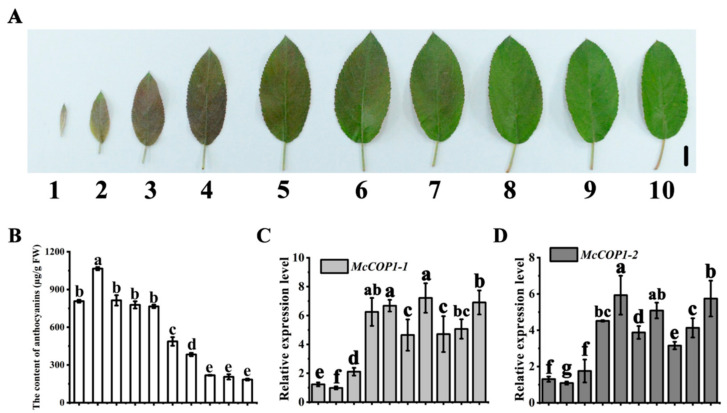
Anthocyanin abundance and transcript levels of *McCOP1* during crabapple leave development. (**A**). Phenotypes of leaves at different growth stages of crabapple. The numbers 1–10 correspond to different leaf growth stages. (**B**). Anthocyanin content in crabapple leaves from the ten developmental stages, in μg/g fresh weight (FW). (**C**). Relative *McCOP1-1* expression levels in leaves from the 10 developmental stages of crabapple were determined using RT-qPCR. (**D**). Relative *McCOP1-2* expression levels in leaves from the 10 developmental stages of crabapple were determined using RT-qPCR. Bar = 1 cm. Real time (RT)-quantitative (q)PCR and high-pressure liquid chromatography (HPLC) were performed with three biological replicates. Error bars indicate the standard error of the mean ± SE of three replicate measurements. Different letters above the bars indicate significantly different values (*p* < 0.05), calculated using one-way analysis of variance (ANOVA) followed by a Tukey’s multiple range test. Scale bar = 1 cm.

**Figure 2:**
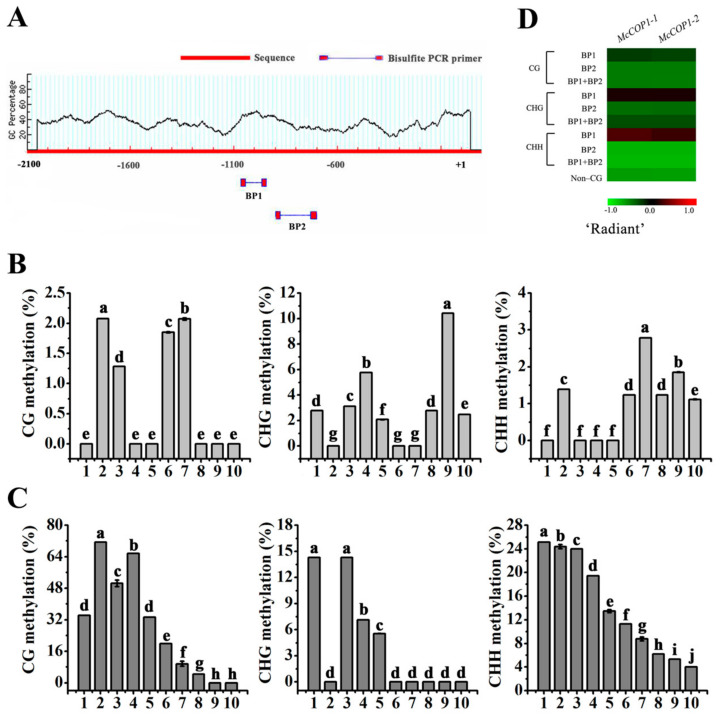
*McCOP1* promoter methylation level in crabapple leaves. (**A**). The positions of BP1 and BP2 primers in the *McCOP1* promoter. (**B**). *McCOP1* promoter methylation level determined using the BP1 primers. (**C**). *McCOP1* promoter methylation level determined using the BP2 primers. The numbers 1–10 represent different leaf growth stages. (**D**). Correlation between the *McCOP1* promoter methylation levels and *McCOP1* gene RNA expression profiles in crabapple leaves visualized as a heat map. Methylation assays were performed with three biological replicates. Error bars indicate the standard error of the mean ± SE of three replicate measurements. Different letters above the bars indicate significantly different values (*p* < 0.05), calculated using one-way analysis of variance (ANOVA) followed by a Tukey’s multiple range test.

We measured anthocyanin levels in ten developmental stages of ‘Radiant’ leaves by high-performance liquid chromatography (HPLC) and observed a gradual decrease (Figure 1A,B). To determine whether methylation of the *McCOP1* promoter might play a part in regulating anthocyanin accumulation, we use two PCR primer pairs (BP1 and BP2) to detect the methylation level of the *McCOP1* promoter (Figure 2A). The results showed that CG, CHG, and especially CHH methylation declined during leaf development (Figure 2B,C).

Two *McCOP1* sequences, *McCOP1-1* and *McCOP1-2* which are paralogs, were cloned from ‘Radiant’ leaves based on homologous sequences in the apple (*M. domestica*) mRNA database (https://www.rosaceae.org/, accessed on 26 July 2021). The full-length coding sequences (1941 bp and 1872 bp, respectively) (Supplementary Figure S1) showed a high degree of nucleotide sequence identity (98.4%) (Supplementary Figure S1). Real time (RT)-quantitative (q)PCR analysis revealed a gradual increase in the expression of both genes during leaf development (Figure 1C). Furthermore, a correlation analysis showed that the degree of CG, CHG and CHH methylation was negatively correlated with *McCOP1-1* and *McCOP1-2* expression, with correlation coefficients of −0.48, −0.43, −0.70 and −0.48, −0.42, −0.70, respectively in *McCOP1-1* and *McCOP1-2* (Figure 2D; Supplementary Table S2). These results suggest that *McCOP1* promoter methylation levels affect *McCOP1* transcript levels.

### 2.2. DNA Methylation Levels of the McCOP1 Promoter Change after Treatment with Methylation Inhibitors

**Figure 3:**
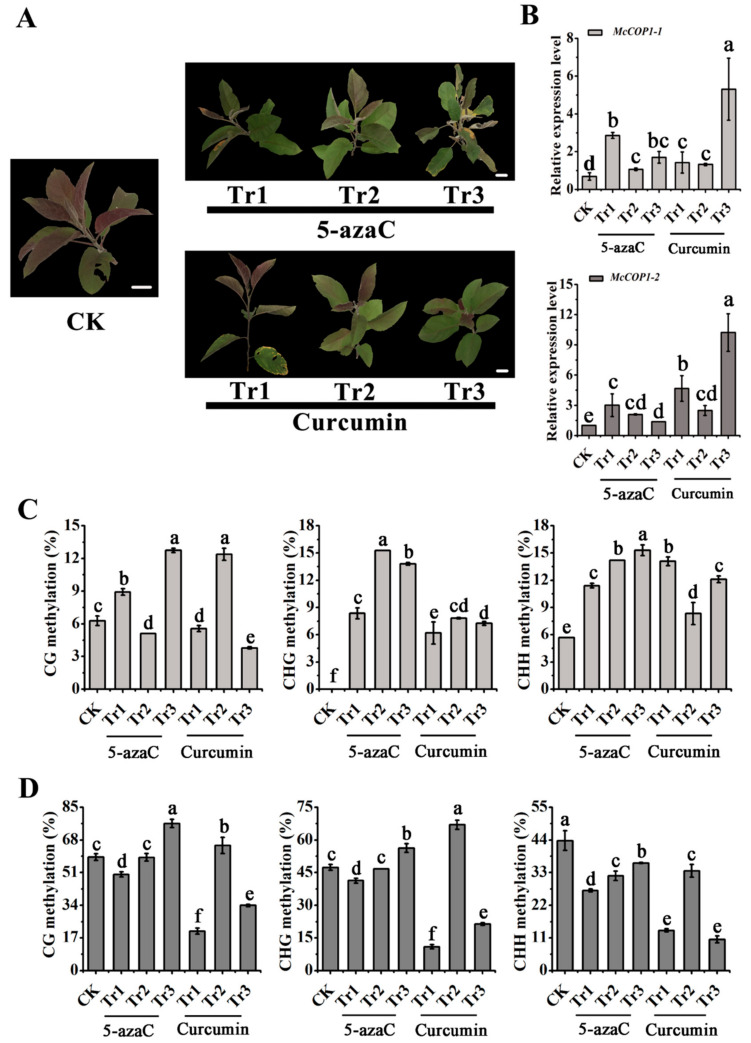
The effect of the 5-azacytidine and curcumin methylation inhibitors on *McCOP1* transcription in ‘Radiant’ leaves. (**A**). Leaf phenotypes after 5-azacytidine (5-azaC) and curcumin treatment. (**B**). Relative *McCOP1-1* and *McCOP1-2* expression. (**C**). *McCOP1* promoter methylation level determined using BP1 primers. (**D**). *McCOP1* promoter methylation level determined using BP2 primers. The concentrations of DNA methylation inhibitors were 0 (CK), 0.1 (Tr1), 1 (Tr2), 10 (Tr3) μmol⋅L^−1^. Bars = 2 cm. Real Time (RT)-quantitative (q) PCR and methylation assays were performed with three biological replicates. Error bars indicate the standard error of the mean ± SE of three replicate measurements. Different letters above the bars indicate significantly different values (*p* < 0.05), calculated using one-way analysis of variance (ANOVA) followed by a Duncan’s multiple range test. Scale bar = 1 cm.

To further confirm that the asymmetric cytosine CHH sites were the main methylation loci in the *McCOP1* promoter, the ‘Radiant’ cultivar was separately treated with the methylation inhibitors 5-azacytidine and curcumin. As shown in Figure 2, as inhibitor concentrations increased, leaf development and coloration were significantly inhibited (Figure 3A). Compared with the control, the relative expression of *McCOP1-1* in leaves increased after 0.1, 1 and 10 μmol·L^−1^ 5-azacytidine or curcumin treatment. We also noted that the methylation inhibitors promoted *McCOP1-2* expression, and that this was especially evident in the 10 μmol·L^−1^ curcumin-treated sample. Overall, the use of DNA methylation inhibitors promoted the expression of the *McCOP1* genes (Figure 3B). In addition, the treatments also resulted in changes in CG, CHH and CHG methylation, and the degree of CHH methylation was significantly lower after treatment, as determined using the BP2 primer set (Figure 3C,D). We therefore concluded that CHH represents the main methylation site in the *McCOP1* promoter.

### 2.3. AGO4 Recognize the SINE Site in the McCOP1 Promoter

In *A. thaliana*, AGO4 is known to be the key RdDM pathway effector, and to regulate the expression levels of target genes by binding to their promoters and mediating promoter methylation [36,37]. To understand how the RdDM pathway affects methylation of the *McCOP1* promoter, we investigated the relationship between the AGO4 protein and the *McCOP1* promoter. DNA fragments corresponding to the coding sequences of *McAGO4*-A (2736 bp), *McAGO4-LIKE* (2811 bp), *McDRM2* (1803 bp) and *McRDM1* (540 bp) were amplified from the leaves of the ‘Royalty’ crabapple cultivar and an alignment of the predicted proteins sequences revealed conserved domains and/or motifs and showed that they were highly similar to the corresponding apple sequences. A phylogenetic analysis, based on predicted amino acid sequences, showed that each crabapple protein grouped to the same branch as the corresponding apple protein (Supplementary Figure S4).

**Figure 4:**
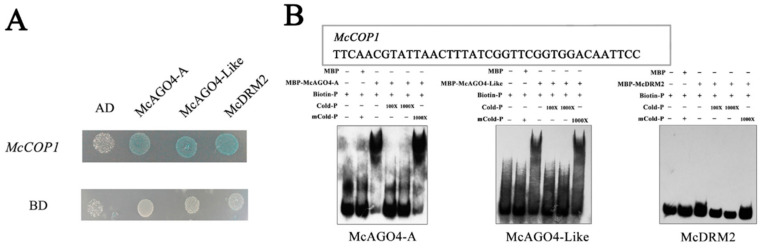
McAGO4 proteins bind to the *McCOP1* promoter. (**A**). Yeast one-hybrid assays indicated that McAGO4-A, McAGO4-Like and McDRM2 bind directly to the *McCOP1* promoter. (**B**). Interaction status of the McAGO4-A, McAGO4-Like, McDRM2 proteins and the *McCOP1* promoter determined by electrophoretic mobility shift assay.

A yeast one-hybrid analysis revealed that McAGO4-A, McAGO4-LIKE and McDRM2 all bind to the *McCOP1* promoter (Figure 4A). We then expressed McAGO4-A, McAGO4-LIKE and McDRM2 in *Escherichia coli* as maltose binding protein (MBP)-tagged recombinant proteins, which were then purified and incubated with DNA probes corresponding to the *McCOP1* promoter, in order to conduct an electrophoretic mobility shift assay (*EMSA*) (Figure 4B). The results suggested that McAGO4 binds to the SINE site (GGTTCGG) in the *McCOP1* promoter. While the McAGO4 proteins bound to the *McCOP1* promoter in both Y2H and EMSA assay, we saw no evidence McDRM2 binding in the EMSA, which indicate false positive results from the yeast one-hybrid assay.

Previous studies using co-immunoprecipitation (co-IP) and pull-down assays have shown that MdAGO4, MdRDM1 and MdDRM2 from apple interact with each other [35]. We also tested the relationship of these three proteins in crabapple using bimolecular fluorescent complementation (BiFC) and confirmed that MdAGO4, MdRDM1 and MdDRM2 can form a protein complex (Supplementary Figure S2). We conclude that RDM1, DRM2 and AGO4 form a complex, and that AGO4 binds promoter of target genes, while RDM1 catalyzes the methylation.

### 2.4. McRDM1 Silencing Inhibits Anthocyanin Biosynthesis in Crabapple

To identify the main factor affecting CHH methylation of the *AtCOP1* (AT2G32950) promoter, we used the *A. thaliana atago4*, *atrdm1* and *atdrm2* mutants. Bisulfite analysis showed that CHH methylation in the *AtCOP1* promoter in leaves of the *atrdm1* mutant was significantly lower compared with the promoter of the other mutants (Supplementary Figure S3). We therefore hypothesized that *RDM1* plays a role in the RdDM pathway.

**Figure 5:**
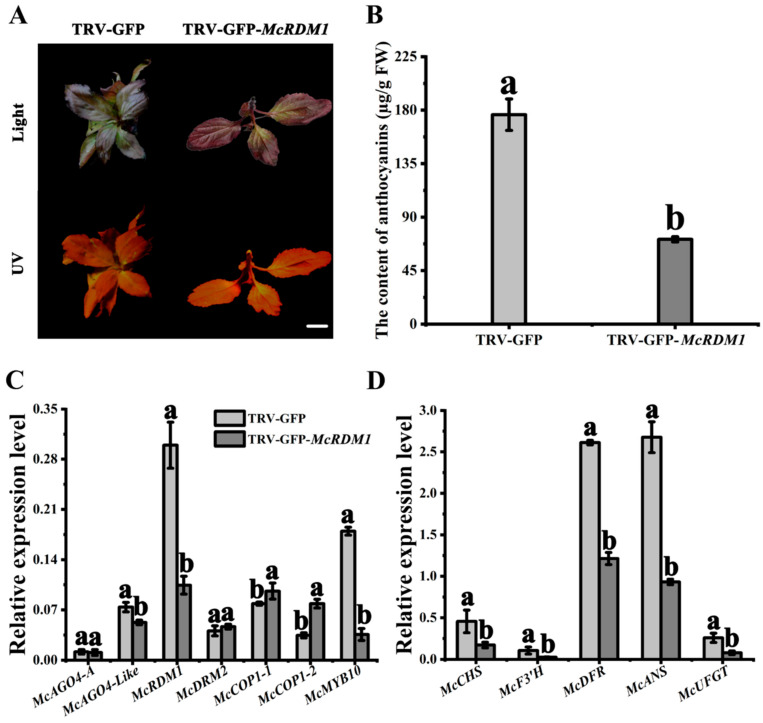
Effects of transient *McRDM1* silencing in ‘Royalty’ leaves. (**A**). Phenotype of *McRDM1*-silenced leaves. (**B**). Anthocyanin contents at the infiltration sites of crabapple leaves in μg/g fresh weight (FW). (**C**). Relative transcript expression levels in leaves around the infiltration sites were determined using Real Time (RT)-quantitative (q) PCR. (**D**). Relative expression levels in crabapple leaves around the infiltration sites were determined using RT-qPCR. Bar = 2 cm. RT-qPCR and HPLC were performed with three biological replicates. Error bars indicate the standard error of the mean ± SE of three replicate measurements. Different letters above the bars indicate significantly different values (*p* < 0.05), calculated using one-way analysis of variance (ANOVA) followed by a Duncan’s multiple range test. Scale bar = 1 cm.

To verify *McRDM1* function and confirm that the RdDM pathway is involved in *McCOP1* promoter methylation and anthocyanin biosynthesis, the *McRDM1* gene was silenced using virus induced gene silencing (VIGS). To this end, the TRV-GFP-*McRDM1* vector was introduced into ‘Royalty’ tissue culture seedlings using vacuum infiltration. Green fluorescence was detected in transgenic seedlings transformed with the TRV-GFP-*McRDM1* and TRV-GFP (empty vector) constructs, and green coloration was seen in the TRV-GFP-*McRDM1*-infected leaves, but not in other plants (Figure 5A). HPLC analysis showed that the anthocyanin levels were substantially lower in the TRV-GFP-*McRDM1* leaves compared to leaves transformed with the empty vector (Figure 5B). Compared with the control, *McRDM1* and *McMYB10* expression, as measured by RT-qPCR, was significantly reduced in *McRDM1*-silenced leaves, while the expression of *McCOP1-1* and *McCOP1-2* increased, and the expression of the anthocyanin synthesis-related genes, *McCHS* (chalcone synthase), *McF3′H* (flavonoid 3′-hydroxylase), dihydroflavonol 4-reductase (*McDFR*), anthocyanidin synthase (*McANS*), and *McUFGT* (UDP-glucose: flavonoid 3-O-glucosyltransferase), were all expressed at lower levels (Figure 5C,D).

**Figure 6:**
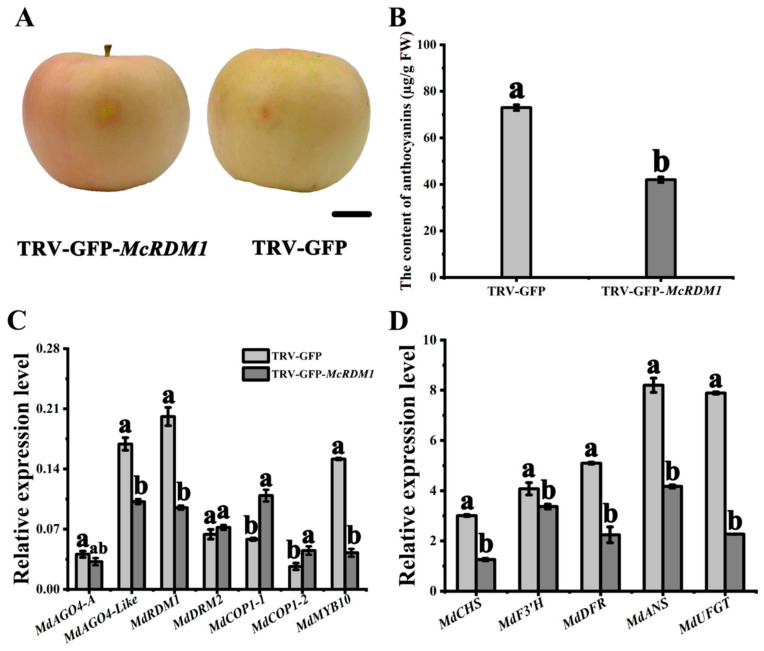
Analysis of transient *McRDM1* silencing in apple fruit. (**A**). The phenotype of infected apple fruits was visualized 4 days post-infiltration. (**B**). Anthocyanin contents at the infiltration sites of apple fruit in μg/g fresh weight (FW). (**C**). Relative expression levels around the infiltration sites were determined using Real Time (RT)-quantitative (q) PCR (RT-qPCR). (**D**). Relative expression levels around the infiltration sites were determined using RT-qPCR. Bar = 2 cm. RT-qPCR and HPLC were performed with three biological replicates. Error bars indicate the standard error of the mean ± SE of three replicate measurements. Different letters above the bars indicate significantly different values (*p* < 0.05), calculated using one-way analysis of variance (ANOVA) followed by a Duncan’s multiple range test. Scale bar = 1 cm.

We also injected apple fruit with an *Agrobacterium tumefaciens* culture containing TRV-GFP-*McRDM1*, and observed fading of the red coloration, as well as lower anthocyanin levels, compared with the empty vector control (Figure 6B). Furthermore, RT-qPCR results showed that the expression of RdDM pathway genes and anthocyanin related genes in these fruit was similar to that in the *McRDM1*-silenced leaves (Figure 6C,D). These results confirm the hypothesis that McRDM1 plays a role in the RdDM complex during *Malus* fruit and leaf coloration.

### 2.5. AtRDM1 Contributes to Anthocyanin Accumulation in A. thaliana under Stress Conditions

**Figure 7:**
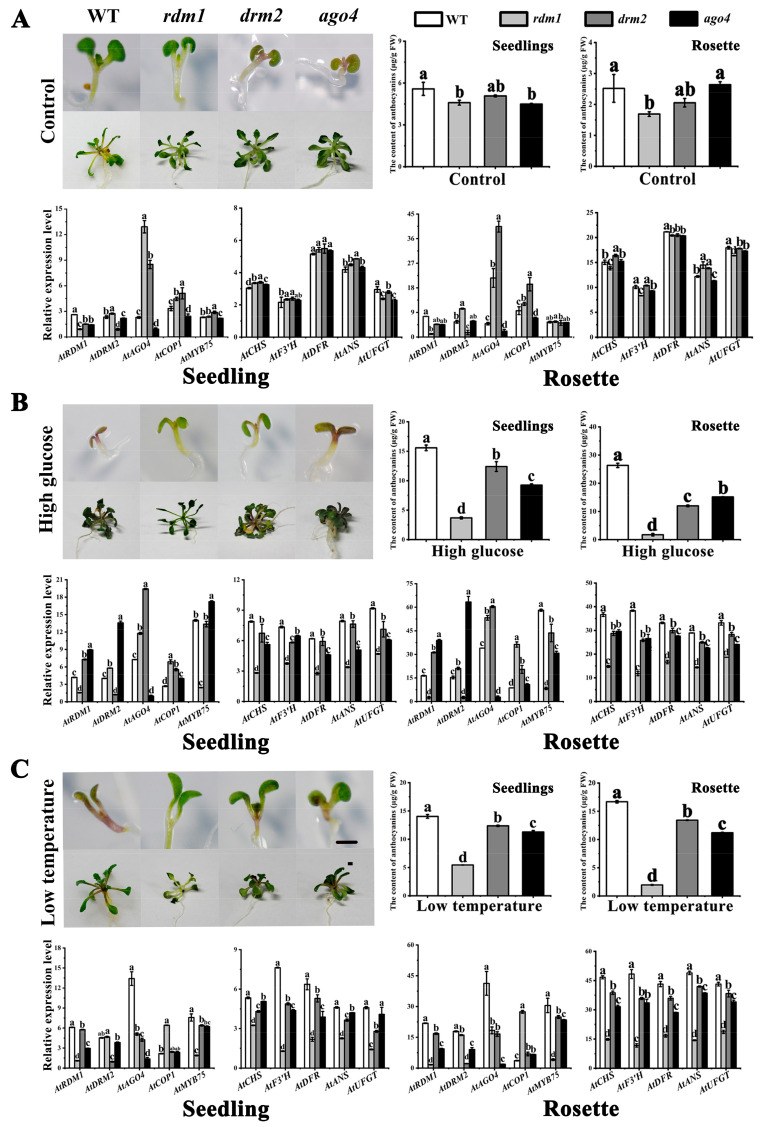
*AtRDM1* affects anthocyanin biosynthesis in *Arabidopsis thaliana* seedlings and rosette leaves in response to stress. (**A**). Phenotype, anthocyanin content and relative gene expression in *A. thaliana* seedlings and rosette leaves in the control group. (**B**). Phenotype, anthocyanin content and relative gene expression in *A. thaliana* seedlings and rosette leaves treated with high glucose. (**C**). Phenotype, anthocyanin content and relative gene expression in *A. thaliana* seedlings and rosette leaves treated with low temperature. Bar = 1 mm. Real Time (RT)-quantitative (q) PCR (RT-qPCR) and HPLC were performed with three biological replicates. Error bars indicate the standard error of the mean ± SE of three replicate measurements. Different letters above the bars indicate significantly different values (*p* < 0.05), calculated using one-way analysis of variance (ANOVA) followed by a Duncan’s multiple range test.

Anthocyanins have been shown to confer a degree of tolerance to abiotic stress, such as low temperature, low nitrogen, high salinity, and drought stress [38,39], and some abiotic stresses have also been reported to affect the RdDM pathway [40]. Since silencing *McRDM1* in crabapple seedlings enhanced anthocyanin accumulation and *McRDM1* and *AtRDM1* have similar functions in the RdDM pathway, we next measured the expression of methylation-related and anthocyanin-related genes in *A. thaliana* mutant seedlings grown under low temperature and high glucose conditions. In response to these treatment, the petioles and leaves of the wild type, and the *atdrm2* and *atago4* mutants became red, while there was no color difference in the *atrdm1* mutant when grown under low temperature or high glucose stresses (Figure 7). HPLC analysis indicated that the anthocyanin accumulation in the *atrdm1* mutant was significantly lower than in wild type, or in the *atdrm2* and *atago4* mutants under low temperature or high glucose stresses. Using RT-qPCR, we also detected a decrease in the transcription of anthocyanin biosynthetic genes in the *atrdm1* mutants (Figure 7). We therefore concluded that the *atrdm1* mutant has impaired anthocyanin accumulation under the imposed stresses, and that *RDM1* promotes anthocyanin accumulation under stress conditions. Expression analysis further showed that *AtCOP1* expression in *atrdm1* increased significantly under low temperature or high glucose stress compared to the control, while the expression of *AtMYB75* decreased after the stress treatments. We observed no significant difference between the *atdrm2* and *atago4* mutants and WT in the expression level of anthocyanin related genes (Figure 7). The same result was also found in *A. thaliana* mutant rosette leaves s (Figure 7).

## 3. Discussion

DNA methylation is known to strongly affect chromatin structure and silence gene expression in both plants and mammals, and it is known that epigenetic modifications can cause somatic mutations in plants [41], including some resulting in color alterations. In floral tissues of the orchid *Oncidium*, the cytosine loci of the *OgCHS* 5′-upstream promoter are methylated, leading to a decrease in *OgCHS* expression and lower levels of anthocyanins [42], and in ‘Max Red Bartlett’ pear, a correlation between *PcMYB10* promoter hypermethylation and a green skin phenotype suggested that DNA methylation silenced expression of the *PcMYB10* transcription factor, resulting in lower anthocyanin abundance [43]. In apple fruit, it has been shown that the MdAGO4 proteins MD07G1052200 and MD07G1052400 interact with MdRDM1 (MD16G1197500) and MdDRM2 (MD17G1031900 and MD09G1029900), forming a protein complex [35]. Moreover, MdAGO4 was found to recognize and bind to the *MdMYB1* promoter sequence containing the ATATCAGA site responsible for CHH methylation to promote anthocyanin biosynthesis [35]. Thus, there are multiple lines of evidence suggesting that DNA methylation plays an extensive role in anthocyanin biosynthesis.

In *Malus* spp., *MYB10* functions to promote anthocyanin biosynthesis by regulating the expression of anthocyanin biosynthesis genes [44,45]. The ubiquitin E3 ligase, *MdCOP1* affects apple fruit coloration by causing ubiquitination and degradation of *MdMYB1* via the proteasome pathway [17]. Additionally, *McMYB10* modulates its own expression by regulating *McCOP1-1* and *McCOP1-2* expression to coordinate McCOP1-mediated ubiquitination of McMYB10 [46]. In this study, we found that CHH methylation in the *McCOP1* promoter negatively correlated with the expression of *McCOP1* during ‘Radiant’ leaf development, and that CHH is the main methylation site in the *McCOP1* promoter. These results provide new insights into the mechanisms by which anthocyanin biosynthesis is regulated.

RDM1, a key effector in the RdDM pathway, can form complexes with AGO4 and DRM2 to jointly regulate gene transcription and physiological development [22]. Mutations in the *RDM1* gene were reported to damage the generation of 24-nucleotide siRNAs, resulting in a decrease in DNA methylation, and a consequent reduction in transcriptional gene silencing at the RdDM target loci [22]. In addition, RDM1 has a role in linking siRNA production with pre-existing or de novo cytosine methylation [22]. Of the two complexes involved in the RdDM pathway that promote the accumulation of siRNAs, RDM1 forms a dimer, which interacts with DRD1 and meristem silencing 3 (DMS3), resulting in a complex with a stoichiometry of 1 DRD1:4 DMS3:2 RDM1 [47]. Moreover, the ability to form homodimers is essential for RDM1 to function fully in the RdDM pathway, which is particularly important during the de novo methylation step [48]. These results are consistent with RDM1 playing a major role in methylation through the RdDM pathway, and our study showed a significant reduction in CHH methylation in the *AtCOP1* promoter in *atrdm1* mutants. Furthermore, anthocyanin accumulation was significantly reduced in crabapple and apple in which *McRDM1* had been transiently silenced, indicating the importance of RDM1 in anthocyanin biosynthesis in *Malus* plants. Finally, the *atrdm1* mutant showed less induction of anthocyanin accumulation under low temperature and high glucose conditions than did wild type and other mutant plants, consistent with RDM1 regulating plant anthocyanin biosynthesis through the RdDM pathway under stress.

Twenty four-nucleotide small interfering RNA (siRNAs) can bind to the effector protein AGO4 and direct de novo DNA methylation by the methyltransferase DRM2 [49,50,51,52]. There are ten AGO proteins (AGO1-AGO10) in *A. thaliana*, of which AGO4, AGO6 and AGO9 act in the RdDM pathway [1,53], and AGO4 in particular has been extensively studied. In plants, the maintenance of CHH methylation is controlled by DRM2, a domain-rearranging methyltransferase from the RdDM pathway [54]. In thi pathway, AGO4 can bind two classes of non-coding RNAs: small interfering RNAs (siRNAs) or long non-coding RNA (lncRNA), to generate complexes that can guide DRM2 to specific genomic locations [55]. Nonsymmetrical CHH (where H is a base other than G) methylation is thought to require de novo methylation by DRM2 after AGO4 binding to chromatin [56]. In *A. thaliana*, AGO4 binding sites are often located distant from genes that are differentially expressed in the *atago4* mutant [57], and it has been shown by chromatin immunoprecipitation (ChIP)-sequencing that AGO4 preferentially binds to a region between 200 to 500 bp upstream of target gene transcription start sites [28]. Recently, a study also showed that MdAGO4 proteins can directly bind to the *MdMYB1* promoter through the ATATCAGA sequence in vivo [35]. In this study, we found that McAGO4 can bind to the *McCOP1* promoter at the SINE site (GGTTCGG), but not the above mentioned ATATCAGA site, suggesting that there are several AGO4 binding sites, and that they lack conservation among the target genes. We here provide evidence of binding by McAGO4 to the promoter of *McCOP1* and the formation of AGO4-RDM1-DRM2 complexes in crabapple leaves.

Plants have evolved mechanisms to promote anthocyanin biosynthesis in response to variation in environmental conditions, such as long day, low temperature and low pH conditions [58,59,60]. Recent studies have suggested that *M. domestica* BTB/POZ and TAZ domain-containing protein 2 (*MdBT2*) negatively regulate hormonal and environmental signal-induced anthocyanin biosynthesis in apple fruit [61]. Under drought conditions, the ethylene response factor ERF family protein 38 (*MdERF38*) interacts with the positive anthocyanin biosynthesis modulator *MdMYB1* and facilitates its binding to anthocyanin biosynthesis genes [62]. Additionally, the apple transcription factor bHLH3 (*MdbHLH3*), has been identified as a positive regulator of cold tolerance and anthocyanin biosynthesis due to its activation of C-repeat-binding factor 2 (*MdCBF2*) and Dihydroflavonol 4-reductase (*MdDFR*) expression [63]. Some abiotic stresses have also been reported to affect methylation and demethylation in plants. For example, salt stress enhances DNA methylation in poplar (*Populus*), than in wild type plants grown under normal conditions involving histone *H3* and 5.8 rDNA loci [64]. In *A. thaliana*, heat stress induces DRM2, DNA-directed RNA polymerase IV subunit 1 (NRPD1) and DNA-directed RNA polymerase V subunit 1 (NRPE1) expression, demonstrating that the RdDM pathway is mobilized by high temperature to strengthen stabilization of DNA methylation [40]. Multiple studies have shown that DNA methylation is also involved in stress responses leading to anthocyanin accumulation. As an example, peach (*Prunus persica*) stored at low temperatures were reported to show higher transcript levels of genes involved I anthocyanin biosynthesis, as well as the transcription factor *PpbHLH3*, and this was associated with lower methylation levels in the promoters of these genes [65]. Such results indicate that plants promote anthocyanin accumulation by regulating promoter methylation under stress conditions.

Here, we report that the RdDM pathway regulates anthocyanin biosynthesis through the formation of an AGO4s (McAGO4-A and McAGO4-like)-RDM1-DRM2 complex, and that McAGO4 binds to the promoter of *McCOP1* in crabapple leaves. We propose that RDM1 is the main effector mediating DNA methylation during stress-induced anthocyanin accumulation.

## 4. Materials and Methods

### 4.1. Plant Materials and Growth

Eight-year-old trees (*Malus* cv ‘Radiant’) were grafted onto *Malus hupehensis* and planted at the Crabapple Germplasm Resources Nursery at the Beijing University of Agriculture (40.1° N, 116.6° E). Young leaves of the ‘Radiant’ cultivar used in this study are red and turn green during maturation. One tree of each cultivar showing consistent growth was selected. Leaf samples were collected from annual branches growing on the fringe of the canopy and in the same compass directions. Leaves of ‘Radiant’ were collected at 10 developmental stages (3, 6, 9, 12, 15, 18, 21, 24, 27 and 30 days after budding), numbered 1–10. All samples were frozen in liquid nitrogen and stored at −80 °C. Branch samples of ‘Radiant’ (15–20 cm) were chosen from annual branches with no germinating buds growing on the fringe and in the same compass directions, and used for methyltransferase inhibitor treatment assays. The *atago4*, *atrdm1* and *atdrm2* single mutants were generated by backcrossing into the *A. thaliana* (Ler) wild type. Plants were obtained from the Beijing University of Agriculture and grown under long day (LD) conditions (16 h: 8 h = light: dark; 20 °C).

### 4.2. Anthocyanin Quantification

Leaf and fruit samples (1.0 g fresh weight) were extracted with 10 mL extraction solution (methanol: water: formic acid: trifluoroacetic acid = 70:27:2:1) at 4 °C in the dark for 72 h, shaking every 6 h. The mixture was then centrifuged at 4 °C at 12,000× *g* for 15 min and the supernatant filtered through a 0.22 μm MilliporeTM filter (Billerica, MA, USA). HPLC analysis was performed as previously described [66], with detection at 520 nm [67]. All samples were analyzed in triplicate (extracted from three different pools of leaves).

### 4.3. Amplification of the McAGO4-A, McAGO4-Like, McDRM2 and McRDM1 and the McCOP1 Promoter

The full-length crabapple *McAGO4-A*, *McAGO4-Like*, *McDRM2* and *McRDM1* gene seqeunces were amplified by PCR using gene-specific primers designed based on GenBank sequences (XM_008377181.1, XM_008365331.1, XM_008381012.1 and ACYM01111433.1). A clone containing a partial cDNA and upstream *McCOP1* promoter sequence was amplified from a DNA template, with a pair of primers designed based on the apple (*M. domestica*) GenBank sequence (AB668570.10). PCR conditions were 5 min at 95 °C followed by 35 cycles of 94 °C for 30 s, 62 ± 2 °C for 30 s, and extension at 72 °C for 2 min. All primers used are listed in Supplementary Table S1.

### 4.4. Measurement of Methylation Levels

We measured the level of methylation through BSP (Bisulfite Sequencing PCR) analysis [68]. A DNA Bisulfite Conversion Kit (TIANGEN BIOTECH, Beijing, China) was used to purify the bisulfite-treated genomic DNA. Digested DNA was used in a Methylation-Specific PCR (TIANGEN BIOTECH, Beijing, China) reaction following the manufacturer’s instructions. The methylation level of each fragment was calculated. Primer sequences are listed in Supplementary Table S1.

### 4.5. Expression Analysis

RT-qPCR was performed with the SYBR^®^ Premix Ex Taq TM II (Takara, Ohtsu, Japan) in a CFX96TM Real-Time PCR System (BIO-RAD, Hercules, CA, USA). Relative transcript levels were quantified by normalizing to the 18S ribosomal RNA gene (*M. domestica*, GenBank DQ341382) using the 2^−ΔΔCT^ method [69]. RT-qPCR analysis was performed with three technical replicates and three biological replicates. Primers are listed in Supplementary Table S1.

### 4.6. Yeast One-Hybrid Assay

The full-length *McAGO4-A*, *McAGO4-Like* and *McDRM2* sequences were cloned into the pJG4-5 (pB42AD) vector [70] for protein expression, and the *McCOP1* promoter sqeunce was ligated into the placZi vector [70]. Constructs were co-transformed into yeast strain EYG48 and selected on a SD/Trp-Ura plate at 28 °C. Yeast culture solutions were used for the color reaction on a SD/Trp-Ura plate (with X-gal added) at 28 °C. All transformations and screenings were performed three times. The primer sequences used are listed in Supplementary Table S1.

### 4.7. EMSA

The full-length *McAGO4-A*, *McAGO4-Like* and *McDRM2* cDNA PCR products were subcloned into the pMAL-C2X expression vector [71]. Bacterial growth and protein induction were performed as described by the manufacturer (Novagen). Following induction of protein expression in the cells with 0.3 mM/100 mL isopropyl-β-D-thiogalactoside (IPTG), and washing with and suspending in PBS solution, 5 mL bacterial aliquots (12 mg/mL) were stored at −80 °C.

Oligonucleotides were synthesized and biotin-labeled at the 3′ end (Sangon Biotech; www.sangon.bioon.com.cn, accessed on 26 July 2021). Standard reaction mixtures [72] were used for EMSA: 2 μL 10 × binding buffer, 1 μL 50% glycerol, 1 μL 100 mM MgCl_2_, 1 μL 1% NP-40, 2 μg protein, 2 μL biotin-labeled oligonucleotides (0.1μM). The reaction mixtures were incubated at room temperature (25 °C) for 20 min and then electrophoretically separated on 12% native polyacrylamide gels and transferred to an Amersham Hybond^TM^ N^+^ nylon membrane (GE Healthcare) in TBE buffer [72] at 380 mA at 4 °C for 40 min. After UV cross-linking, biotin-labeled DNA was detected using a LightShift Chemiluminescent EMSA kit (Pierce, Shanghai, China). The primer and probe sequences are listed in Supplementary Table S1.

### 4.8. BiFC

The *McAGO4-A, McAGO4-Like, McDRM2*, *McRDM1*, *MdCOP1-1*, *MdCOP1-2* and *McMYB10* coding sequences were cloned into the p35SYCE and p35SYNE vectors, which contain DNA encoding the C-terminal or N-terminal regions of yellow fluorescent protein (YFP) (YFPN or YFPC), respectively. Tobacco (*Nicotiana benthamiana*) leaves were transiently co-transformed using an *A. tumefaciens* (GV3101) infection method with different combinations of these constructs [73]. YFP-dependent fluorescence was detected 24 h after transfection using a confocal laser-scanning microscope (Zeiss LSM 510 Meta). All images correspond to single optical slices of epidermal cells. Excitation and emission wavelengths were 514 and 527 nm, respectively, for enhanced YFP. Specific primers are listed in Supplementary Table S1.

### 4.9. RDM1 Silencing in Crabapple Plantlets and Apple Fruit

The *McRDM1* full-length cDNA was designed with *Xba*I and *BamH*I sites for insertion into the TRV2 vector, which was transformed into *A. tumefaciens* (GV3101) and used for transient co-transformation with the TRV1 vector into cv ‘Royalty’ seedlings and ‘Red Fuji’ fruit via vacuum infiltration and injection, respectively [67]. A handheld UV instrument (SUNLONGE SUPER UV LED LAMP) was to detect and record the GFP fluorescence of the successfully transformed apple seedlings and fruit 5 days after transformation for seedlings and 3 days for fruit [67]. Plant leaves showing GFP fluorescence were used to detect gene expression levels and anthocyanin content after UV detection.

## Supplementary Materials

The following are available online at https://www.mdpi.com/article/10.3390/plants10112466/s1, Figure S1: Cloning and sequence characterization of *McCOP1-1* and *McCOP1-2*. A. *McCOP1* nucleotide sequence alignment. B. Location of *McCOP1* coding sequences on chromosome 10. The sequence in the box is the 69-bp deletion in the *McCOP1-1* gene. Blue and green font represent the Real time (RT)-quantitative (q)PCR primers for *McCOP1-1* and *McCOP1-2*, respectively. Abbreviations: Md, *Malus x domestica* (AB668570.1); Rosa, *Rosa* spp. *hybrid cultivar* (AF394913.1). Figure S2: Interaction between McRDM1, McRDM2, and McAGO4. Bimolecular fluorescent complementation (BiFC) assay using *Nicotiana benthamiana*. Fusion constructs containing partial yellow fluorescent protein sequences and McRDM1/McDRM2, McRDM1/McAGO4-A and McRDM1/McAGO4-like were transiently co-expressed in *Nicotiana benthamiana* leaves. The yellow fluorescence represents protein interactions. Figure S3: *AtCOP1* promoter methylation level determined by bisulfite sequencing using different primers. A. The phenotype of WT, *atago4*, *atdrm2* and *atrdm1* seedlings. B. The position of BP3 and BP4 primers in the promoter sequence. C. *AtCOP1* promoter methylation level using BP3 primers. D. *AtCOP1* promoter methylation level using BP4 primers. Error bars indicate the standard error of the mean ± SE of three replicate measurements. Different letters above the bars indicate significantly different values (*p* < 0.05) calculated using one-way analysis of variance (ANOVA) followed by a Duncan’s multiple range test. Figure S4: Sequence analysis of McAGO4-Like, McAGO4-A, McDRM2 and McRDM1. A. Amino acid sequence alignment. B. Phylogenetic analysis of McCOP1 proteins from different plant species and functional domain analysis. The alignment was conducted with the Mega10 program using ClustalX. The abbreviations used are as follows: Md, *Malus x domestica*; Pp, *Prunus persica*; Pm, *Prunus mume*; Fv, *Fragaria vesca* spp.; Vv, *Vitis vinifera*; At, *Arabidopsis thaliana*. Table S1: Primer sequences used in this study. Table S2: Correlation analysis between the *McCOP1* promoter methylation level and *McCOP1* RNA expression profiles in ‘Radiant’ leaves.
