# Supplementary material for: The RNA Directed DNA Methylation (RdDM) Pathway Regulates Anthocyanin Biosynthesis in Crabapple (Malus cv. spp.) Leaves by Methylating the McCOP1 Promoter
Source: Plants (Basel). 2021 Nov 15;10(11):2466. doi: 10.3390/plants10112466 (PMC8618851; doi:10.3390/plants10112466)
Supplement: Supplementary file 1 [file plants-10-02466-s001.zip › plants-1335915-supplementary.pdf]

Supplementary Figures

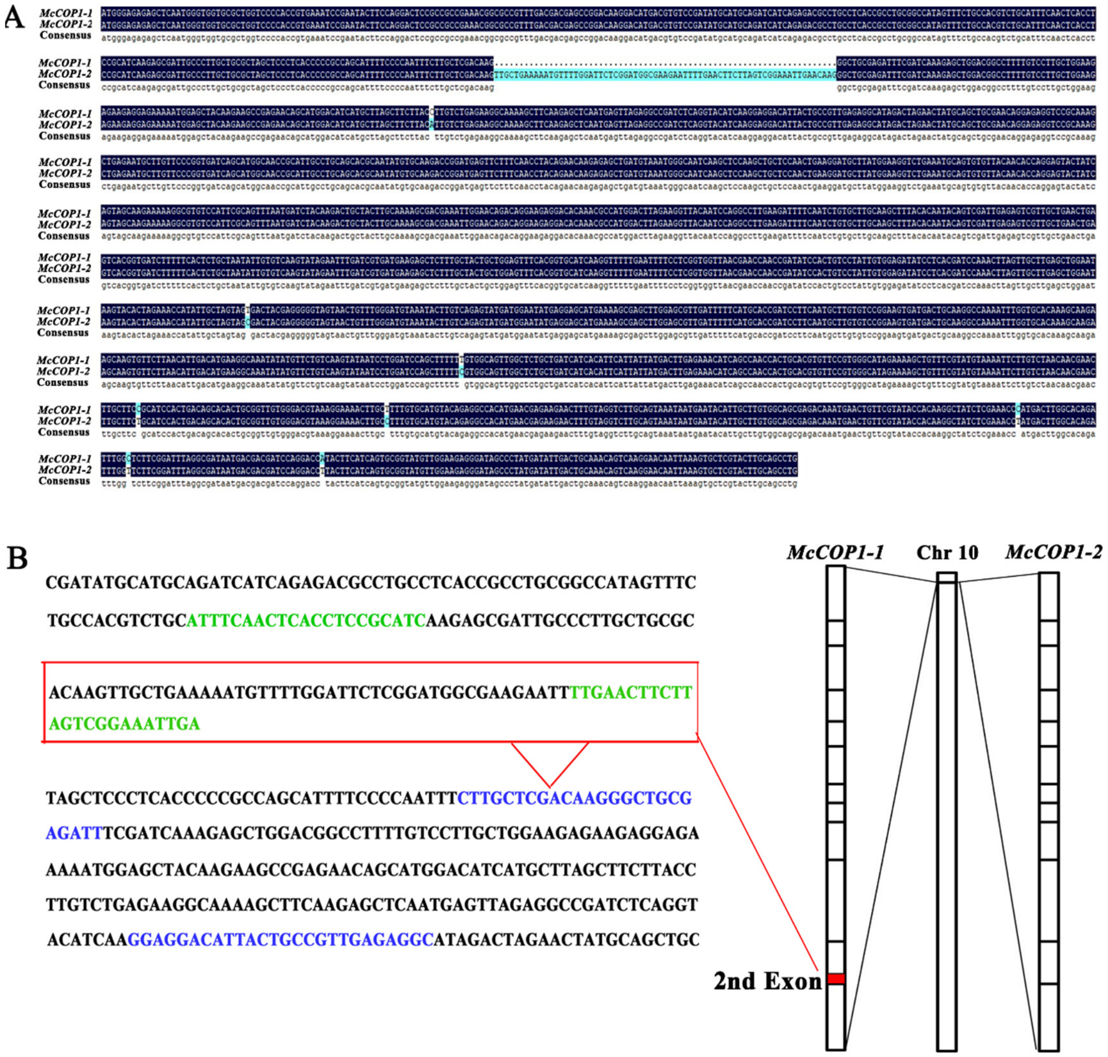

Supplementary Figure S1. Cloning and sequence characterization of *McCOP1-1* and *McCOP1-2*. A. *McCOP1* nucleotide sequence alignment. B. Location of *McCOP1* coding sequences on chromosome 10. The sequence in the box is the 69-bp deletion in the *McCOP1-1* gene. Blue and green font represent the Real time (RT)-quantitative (q)PCR primers for *McCOP1-1* and *McCOP1-2*, respectively. Abbreviations: Md, *Malus x domestica* (AB668570.1); Rosa, *Rosa* spp. *hybrid cultivar* (AF394913.1).

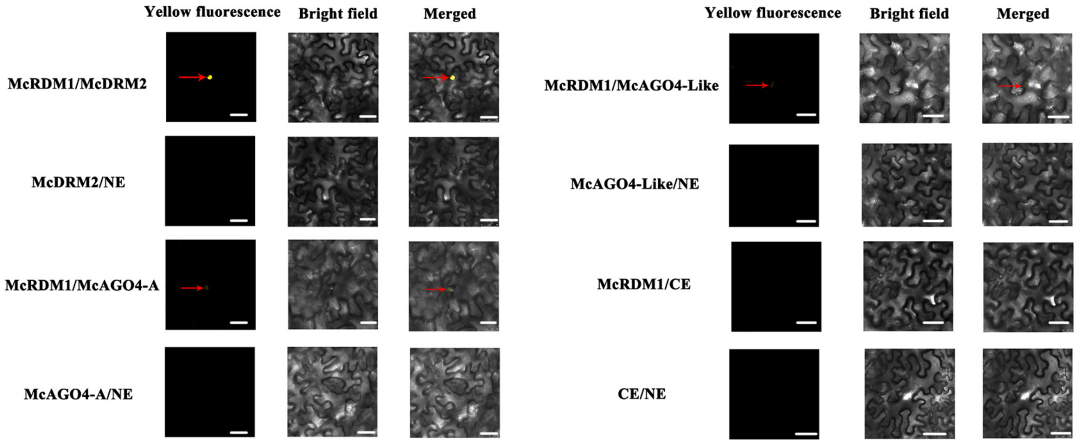

Supplementary Figure S2. Interaction between McRDM1, McRDM2, and McAGO4. Bimolecular fluorescent complementation (BiFC) assay using *Nicotiana benthamiana*. Fusion constructs containing partial yellow fluorescent protein sequences and McRDM1/McDRM2, McRDM1/McAGO4-A and McRDM1/McAGO4-like were transiently co-expressed in *Nicotiana benthamiana* leaves. The yellow fluorescence represents protein interactions. Scale bar=20 μm.

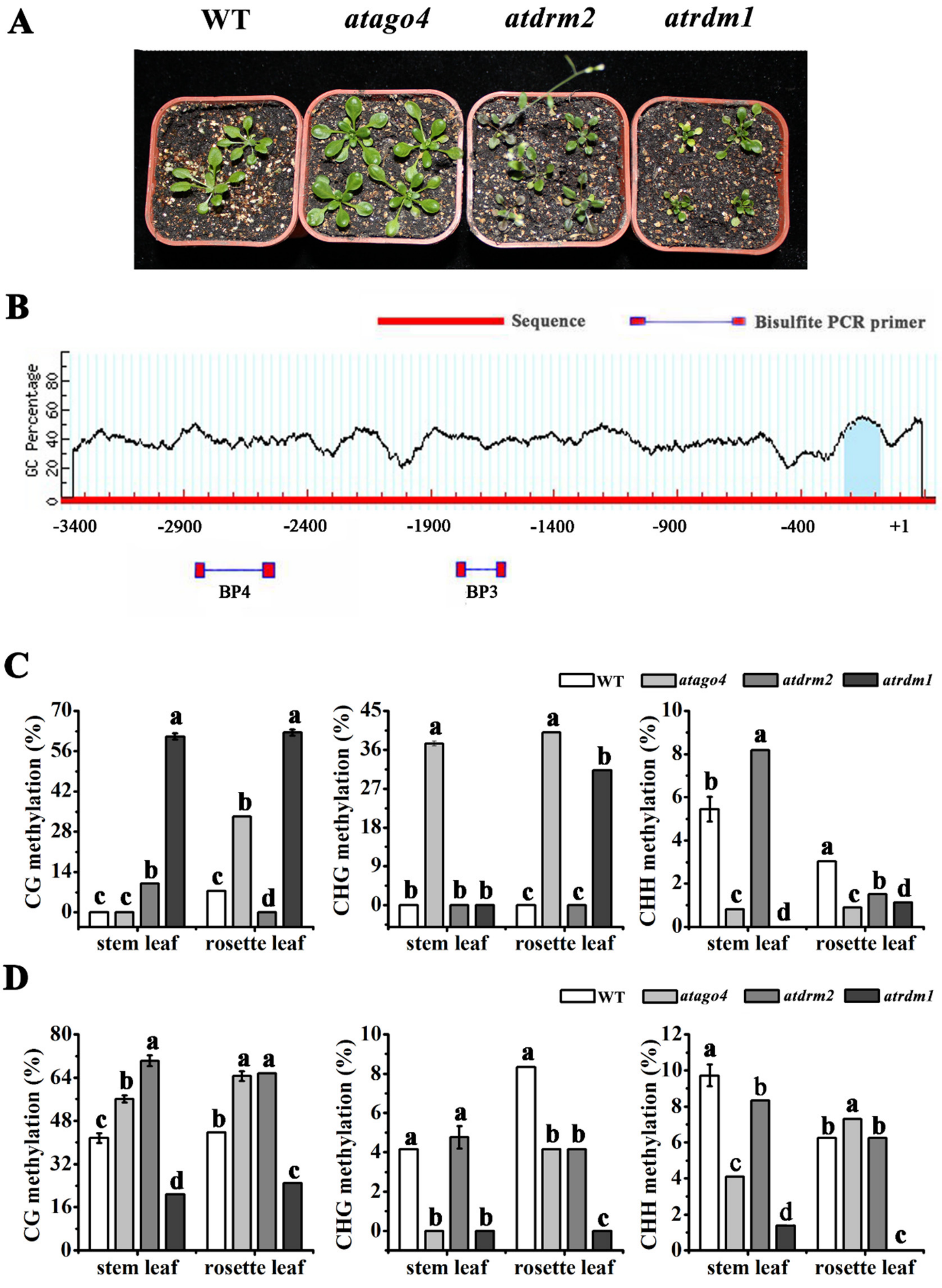

Supplementary Figure S3. *AtCOP1* promoter methylation level determined by bisulfite sequencing using different primers. A. The phenotype of WT, *atago4*, *atdrm2* and *atrdm1* seedlings. B. The position of BP3 and BP4 primers in the promoter sequence. C. *AtCOP1* promoter methylation level using BP3 primers. D. *AtCOP1* promoter methylation level using BP4 primers.

Error bars indicate the standard error of the mean ± SE of three replicate measurements. Different letters above the bars indicate significantly different values ($p < 0.05$) calculated using one-way analysis of variance (ANOVA) followed by a Duncan’s multiple range test.

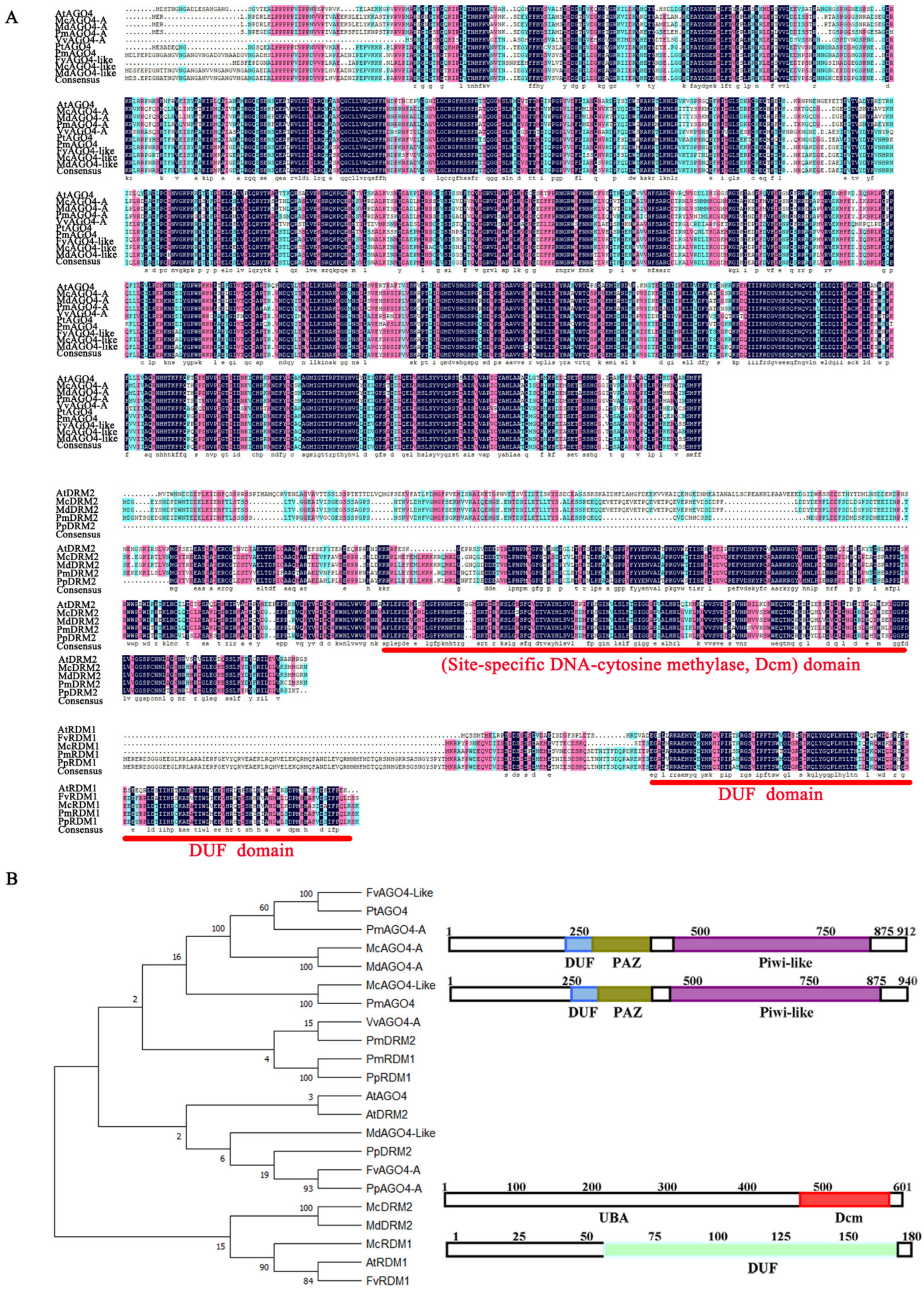

Supplementary Figure S4. Sequence analysis of McAGO4-Like, McAGO4-A, McDRM2 and

McRDM1. A. Amino acid sequence alignment. B. Phylogenetic analysis of McCOP1 proteins from different plant species and functional domain analysis. The alignment was conducted with the Mega10 program using ClustalX. The abbreviations used are as follows: Md, *Malus x domestica*; Pp, *Prunus persica*; Pm, *Prunus mume*; Fv, *Fragaria vesca* spp.; Vv, *Vitis vinifera*; At, *Arabidopsis thaliana*.

Supplementary Tables

Supplementary Table S1. Primer sequences used in this study

Primer	Oligonucleotide sequence 5' -> 3'
**Cloning**	
Q-McCOP1-F	GAAGTATTCGGATTTCACGGTGGGGAC
Q-McCOP1-R	GACCTCCCAATTCTCGAAGGCCAAA
McCOP1-F	TCACGCACGCTTCACCCAGA
McCOP1-R	TGTAGCGATTCAAGACGGGGTT
McAGO4-A-F	GGCTTGTGGTTGAGCAGGTCTTAG
McAGO4-A-R	CGGCATCGAGGGATAAGAAACTGAAG
McAGO4-Like-F	CACGGCTGGATTTCAGAAGCGGTT
McAGO4-Like-R	GGTAACCATAGTAGGGCTCAAGGCTC
McDRM2-F	GGAGCTTGTAAATCGCTGCGTGGGC
McDRM2-R	CCACAAGTAACCAGCCGACCGCAC
McRDM1-F	GTTTACAATGGATCCCACACG
McRDM1R	CCAAGCATCATAAAACAGTCC
**Y1H**	
YEMcAGO4-A-XhoI-F	AACTCGAGATGGAGCGCTTGAATCCCG
YEMcAGO4-A-NotI-R	TAGCGGCCGCTTAACAGAAGAACATGGAG
YEMcAGO4-Like-XhoI-F	CTCTCGAGATGGATTCGTTTGAGCCAGATG
YEMcAGO4-Like-NotI-R	TAGCGGCCGCTCAACAGAAGAACATG
YEMcDRM2-EcoRI-F	CGGAATTCATGGATGGTGAATACAGTAATG
YEMcDRM2-XhoI-R	TCTCGAGTCAATTGTTTCTTCCCATCATG
YEMcCOP1-EcoRI-F	ACGAATTCGACCTCCCAATTCTCG

YEMcCOP1-XhoI-R	ACCTCGAGGAAGTATTCGGATTTCAC
**BiFC**	
McCOP1s-SalI-F	ACGC GTCGAC ATGGGAGAGAGCTCAATGG
McCOP1s-KpnI-R	CCGG GGTACC GGCTGCAAGTACGAGCAC
McMYB10-SalI-F	ACGC GTCGAC ATGGAGGGATATAACGAA
McMYB10-KpnI-R	CCGG GGTACC TTCTTCTTTTGAATGATTC
McAGO4-A-SalI-F	ACGC GTCGAC ATGGAGCGCTTGAATCCC
McAGO4-A-KpnI-R	CCGG GGTACC ACAGAAGAACATGGAGTTG
McAGO4-Like-SalI-F	ACGC GTCGAC ATGGATTCGTTTGAGCCAG
McAGO4-Like-KpnI-R	CCGG GGTACC ACAGAAGAACATGGAACTG
McDRM2-SalI-F	ACGC GTCGAC ATGGATGGTGAATACAGT
McDRM2-KpnI-R	CCGG GGTACC ATTGTTTCTTCCCATCATGC
McRDM1-SalI-F	CCGC GGATCC ATGAAGAGGGCAGCTCCC
McRDM1-KpnI-R	CCGG GGTACC CGATTTGCTTCGTAGCTGG
**BPS**	
BP1-F	TTGTTTTTTTGATTGATGATTAAAT
BP1-R	TATACATATTTACTCTATTCCTAACTCCTC
BP2-F	TGTGTTTTTGATAAATGTTTTTTTT
BP2-R	AATCTACAATACCTTCACAATCTATTTAAA
BP3-F	ATAAGATTTATTTTGATATTTTGAAA
BP3-R	TACTATTAATCCTCTACCACAAAAC
BP4-F	ATTTTTATTGAAAATTTGGAAAA
BP4-R	CATATAAAAATTAAAACAACACACC
**qRT-PCR**	

qMc/MdCOP1-1-F	ATTTCAACTCACCTCCGCATC
qMc/MdCOP1-1-R	TCAATTTCCGACTAAGAAGTTCAAA
qMc/MdCOP1-2-F	CTTGCTCGACAAGGGCTGCGAGATT
qMc/MdCOP1-2-R	GCCTCTCAACGGCAGTAATGTCCTCC
qMc/MdMYB10-F	GGACCAGCAGCAGGAAACTA
qMc/MdMYB10-R	ACAACCCTCCATTAATGCCGAC
qMc/MdAGO4-A-F	CTCCACCACCACCCATTATTCC
qMc/MdAGO4-A-R	TGCCCTTTAGTTCCATTCCCAC
qMc/MdAGO4-Like-F	CTATGATGGGGAGAAGAGCCTG
qMc/MdAGO4-Like-R	ACAGTTTCCATTGTTCCTGCTTG
qMc/MdDRM2-F	TTTTTTGCGGTTGAAGAGAAGCC
qMc/MdDRM2-R	TGAACTGTCTCATCATCATCCCCT
qMc/MdRDM1-F	ACCTCACCAATCTTCTCCTAAAACA
qMc/MdRDM1-R	TGGCTTCGGCTTTACAAGGATG
qMc/MdCHS-F	GGAGACAACTGGAGAAGGACTGGAA
qMc/MdCHS-R	CGACATTGATACTGGTGTCTTCA
qMc/MdF3’H-F	ACGATGGCGGATGTTACGG
qMc/MdF3’H-R	GCTTTGACCCTGCACTTGCT
qMc/MdDFR-F	GATAGGGTTTGAGTTCAAGTA
qMc/MdDFR-R	TCTCCTCAGCAGCCTCAGTTTTCT
qMc/MdANS-F	GGAGAAGATCATCCTTAAGCCA
qMc/MdANS-R	CTAAGATATATCATACCAACTATGCC
qMc/MdUFGT-F	CCACCGCCCTTCCAAACACTCT
qMc/MdUFGT-R	CACCCTTATGTTACGCGGCATGT

qMc18S-F	ACACGGGGAGGTAGTGACAA
qMc18S-R	CCTCCAATGGATCCTCGTTA
qAtCHS-F	GCCGAGAACAATCGTGGAGCA
qAtCHS-R	AGTCAAGGTGGGTGTCAGAGG
qAtF3'H -F	TGGTAAAAAAGGAGGATTCAT
qAtF3'H-R	CCGTGAGTAGTCTCTGTTTCT
qAtDFR-F	TGGTGTCGGTCCATTCAT
qAtDFR-R	GAGAGAGCGCGGTGATAAGG
qAtUFGT-F	TGGAGGTGGCGGTTGAA
qAtUFGT-R	CTTTGCCGCGAGAACCA
qAtANS-F	AATGGAGCGTGTCAAGAAAGC
qAtANS-R	TTGAATCTTTCCAGTGGCTTG
qAtAGO4-F	ATTGTTACTCAATGCATGGCTC
qAtAGO4-R	GTGTCCGAACAGATGCTCTATA
qAtDRM2-F	ATGGTGATTTGGAATAACGA
qAtDRM2-R	TCAAGATCCTCTCATCCTCG
qAtRDM1-F	CAATGACAATGGAACTACGACC
qAtRDM1-R	ATACATCTCTGCTCTTCTCAGC
qAtCOP1-F	GTTGCTTGAGTTGGAATAAGCA
qAtCOP1-R	AAACTTTAACCTTGCAGTCGTC
qAtMYB75-F	TTCCTGTAAGAGCTGGGCTA
qAtMYB75-R	TTAAAGACCACCTATTCCCT
qAtACTIN-F	CTCTCCCGCTATGTATGTCGCCA
qAtACTIN-R	GTGAGACACACCATCACCAG

Supplementary Table S2. Correlation analysis between the *McCOP1* promoter methylation level and *McCOP1* RNA expression profiles in ‘Radiant’ leaves.

Correlation coefficient values		McCOP1 expression levels		CG methylation			CHG methylation			CHH methylation		
		McCOP 1-1	McCOP 1-2	BP1	BP2	BP1+ BP2	BP1	BP2	BP1+ BP2	BP1	BP2	BP1+ BP2
McCOP1s expression level	McCOP1-1	1	0.95**	-0.24	-0.48**	-0.48**	0.11	-0.43*	-0.31	0.27	-0.70**	-0.72**
	McCOP1-2	0.95**	1	-0.27	-0.48**	0.49**	0.11	-0.42*	-0.31	0.21	-0.70**	-0.72**
CG methylation	BP1	-0.24	-0.27	1	0.26	0.29	-0.63**	-0.23	-0.48**	0.48**	0.23	0.31
	BP2	-0.48**	-0.48**	0.26	1	1.00**	-0.18	0.47**	0.32	-0.53**	0.88**	0.87**
	BP1+BP2	-0.48**	-0.49**	0.29	1.00**	1	-0.2	0.46**	0.3	-0.51**	0.88**	0.88**
CHG methylation	BP1	0.11	0.11	-0.63**	-0.18	-0.2	1	0.09	0.53**	-0.1	-0.21	-0.24
	BP2	-0.43*	-0.42*	-0.23	0.47**	0.46**	0.09	1	0.89**	-0.78**	0.73**	0.69**
	BP1+BP2	-0.31	-0.31	-0.48**	0.32	0.3	0.53**	0.89**	1	-0.71**	0.53**	0.48**
CHH methylation	BP1	0.27	0.21	0.48**	0.53**	-0.51**	-0.1	-0.78**	-0.71**	1	-0.59**	-0.51**
	BP2	-0.70**	-0.70**	0.23	0.88**	0.88**	-0.21	0.73**	0.53**	-0.59**	1	1.00**
	BP1+BP2	-0.72**	-0.72**	0.31	0.87**	0.88**	-0.24	0.69**	0.48**	-0.51**	1.00**	1

* $p<0.05$ ** $p<0.01$
